# Environmental Surveillance for *Salmonella* Typhi and its Association With Typhoid Fever Incidence in India and Malawi

**DOI:** 10.1093/infdis/jiad427

**Published:** 2023-09-29

**Authors:** Christopher B Uzzell, Dilip Abraham, Jonathan Rigby, Catherine M Troman, Satheesh Nair, Nicola Elviss, Lalithambigai Kathiresan, Rajan Srinivasan, Veeraraghavan Balaji, Nicolette A Zhou, John Scott Meschke, Jacob John, Gagandeep Kang, Nicholas Feasey, Venkata Raghava Mohan, Nicholas C Grassly

**Affiliations:** Department of Infectious Disease Epidemiology, Imperial College London, London, United Kingdom; Division of Gastrointestinal Sciences, Christian Medical College, Vellore, India; Malawi-Liverpool Wellcome Programme, Kamuzu University of Health Sciences, Blantyre, Malawi; Department of Infectious Disease Epidemiology, Imperial College London, London, United Kingdom; Gastrointestinal Bacteria Reference Unit, UK Health Security Agency, London, United Kingdom; Science Group, UK Health Security Agency, London, United Kingdom; Department of Community Health, Christian Medical College, Vellore, India; Department of Community Health, Christian Medical College, Vellore, India; Division of Antimicrobial Resistance and Surveillance, Christian Medical College, Vellore, India; Department of Environmental and Occupational Health Sciences, School of Public Health, University of Washington, Seattle, Washington, USA; Department of Environmental and Occupational Health Sciences, School of Public Health, University of Washington, Seattle, Washington, USA; Department of Community Health, Christian Medical College, Vellore, India; Division of Gastrointestinal Sciences, Christian Medical College, Vellore, India; Malawi-Liverpool Wellcome Programme, Kamuzu University of Health Sciences, Blantyre, Malawi; Department of Clinical Sciences, Liverpool School of Tropical Medicine, Liverpool, United Kingdom; Department of Community Health, Christian Medical College, Vellore, India; Department of Infectious Disease Epidemiology, Imperial College London, London, United Kingdom

**Keywords:** *Salmonella* Typhi, typhoid fever, typhoid-paratyphoid vaccines, wastewater surveillance, India, Malawi

## Abstract

**Background:**

Environmental surveillance (ES) for *Salmonella* Typhi potentially offers a low-cost tool to identify communities with a high burden of typhoid fever.

**Methods:**

We developed standardized protocols for typhoid ES, including sampling site selection, validation, characterization; grab or trap sample collection, concentration; and quantitative PCR targeting *Salmonella* genes (*ttr*, *staG,* and *tviB*) and a marker of human fecal contamination (HF183). ES was implemented over 12 months in a historically high typhoid fever incidence setting (Vellore, India) and a lower incidence setting (Blantyre, Malawi) during 2021–2022.

**Results:**

*S.* Typhi prevalence in ES samples was higher in Vellore compared with Blantyre; 39/520 (7.5%; 95% confidence interval [CI], 4.4%–12.4%) vs 11/533 (2.1%; 95% CI, 1.1%–4.0%) in grab and 79/517 (15.3%; 95% CI, 9.8%–23.0%) vs 23/594 (3.9%; 95% CI, 1.9%–7.9%) in trap samples. Detection was clustered by ES site and correlated with site catchment population in Vellore but not Blantyre. Incidence of culture-confirmed typhoid in local hospitals was low during the study and zero some months in Vellore despite *S.* Typhi detection in ES.

**Conclusions:**

ES describes the prevalence and distribution of *S.* Typhi even in the absence of typhoid cases and could inform vaccine introduction. Expanded implementation and comparison with clinical and serological surveillance will further establish its public health utility.

## Teaser

Environmental (wastewater) surveillance for *Salmonella* Typhi using standardized protocols found a higher prevalence in India compared with Malawi, consistent with recent studies of typhoid fever incidence. Further work to establish the public health utility of typhoid environmental surveillance is warranted.

Over the last 30 years, the global incidence of typhoid fever, caused by *Salmonella enterica* serovar Typhi (*S.* Typhi), has halved because of improvements in water and sanitation and widespread use of effective antibiotics. However, there were still an estimated 10.9 million cases and 117 000 deaths globally in 2017, mainly in Africa and south and southeast Asia [1]. Moreover, reductions in disease incidence are at risk of being reversed with the emergence of extensively resistance *S.* Typhi in Asia and increasing detection of strains with reduced susceptibility to the commonly used oral antibiotic, azithromycin [2, 3].

Fortunately, 2 highly effective typhoid conjugate vaccines (TCVs) have recently been prequalified for global use by the World Health Organization (WHO) (in 2017 and 2020, respectively) [4, 5]. WHO recommends the introduction of TCV to control typhoid fever, prioritizing countries with the highest burden of disease or a high burden of antimicrobial-resistant *S.* Typhi, with national decisions on the preferred vaccination strategy (universal, risk-based, or phased) based on local surveillance data [6].

The gold standard for surveillance of typhoid fever is blood culture of suspected cases but such data are severely limited, reflecting the cost and challenges with establishing and sustaining blood culture capabilities in low-resource settings [7]. *S.* Typhi is, however, shed in feces and spread by fecal contamination of food and water [8]. Environmental surveillance (ES), based on testing sewage and wastewater for *S.* Typhi, has therefore been suggested as a low-cost surveillance tool to identify *S.* Typhi circulation and inform national decision-making about the introduction of TCV, and where phased introduction is necessary, enabling highest risk areas to be targeted first [9, 10].

Previous methods employed to detect *S.* Typhi in the environment have varied considerably, including ES site selection, and sample collection, concentration, and laboratory processing [11]. Alternative methods vary in sensitivity to detect *S.* Typhi, making it difficult to interpret different levels of detection in the environment [12]. Several studies have used polymerase chain reaction (PCR) or culture to detect *S.* Typhi in ES wastewater samples in areas where disease is common [13–16]. However, the relationship between the prevalence and abundance of *S.* Typhi detection in ES samples and the incidence of typhoid fever in the local community is unknown.

We developed a set of standardized protocols for typhoid ES, covering each stage of implementation from ES sample site selection, through sample collection to laboratory processing and PCR strategy [17]. We evaluated these protocols, including 2 alternative sampling methods, over 12 months in Vellore, India and Blantyre, Malawi. Here we report a comparison of *S.* Typhi detection in ES samples in the 2 settings, and on the relationship with clinical disease incidence, to evaluate the potential utility of ES as a low-cost tool for typhoid surveillance.

## METHODS

### Study Design

We compared the prevalence of *S.* Typhi in ES samples in a very high typhoid fever incidence community (Vellore, India) with that in a lower incidence community (Blantyre, Malawi). The study areas in Vellore and Blantyre were selected to cover the major urban populations served by hospital-based typhoid disease surveillance. A total of 141 800 people were living in the Vellore study area (16 km^2^) served by the Christian Medical College Hospital and its 2 satellite facilities. The Blantyre study area was considerably larger (214 km^2^) with 489 900 people served by the Queen Elizabeth Central Hospital. To identify a significant difference in the prevalence of *S.* Typhi between the 2 communities, we estimated that we would require monthly samples from approximately 40 ES sites in each community over 1 year. Full details of the study design, study areas, and sample size calculations are provided in the published study protocol [17]. Study protocols covering grab and Moore swab sampling, swab construction, DNA extraction, and qPCR are available at https://www.protocols.io/workspaces/typhoides.

### ES Site Selection

Candidate ES sites (sampling points) were systematically identified at wastewater confluence points using remote geographic information systems analysis based on environmental data [17]. In brief, geospatially referenced “blue-line” data on sewage drainage channels (Vellore) and river systems (Vellore and Blantyre) were integrated with high-resolution digital elevation models to identify candidate ES sites, delineate hydrological catchments, and estimate their associated populations using the High-Resolution Settlement Layer [18]. Sites were selected to represent a range of catchment population sizes across the study area. Once identified, candidate sites were visited by the field team and their suitability for inclusion assessed based on accessibility, safety, and adequate flow (sample site questionnaire).

### Sample Collection and Processing

Monthly grab and trap (Moore swab) samples were collected for 1 year beginning in May 2021 at each ES site according to the study protocols [12, 17]. Moore swabs were deployed 48 hours prior to collection. Grab samples (1000 mL) were then collected at the same time as the Moore swabs, with a recommended collection time between 6 Am and 10 Am. Moore swabs were incubated overnight in universal preenrichment (UPE) broth before filtration (0.45 μm) and storage of the filter disc at −2°C prior to DNA extraction. Grab samples were filtered (0.45 μm) after prefiltering through a coffee filter, with the filters eluted in Ringer's lactate and the eluate centrifuged to generate a pellet for storage at −2°C. Personal protective equipment was worn during sample collection and infection control procedures followed to minimize risks to personnel and the potential for sample contamination.

### DNA Extraction and PCR Strategy

DNA was extracted using the QIAamp Powerfecal Pro kit (Qiagen), eluted into 50 μL and stored at −20°C. Multiplex quantitative PCR (qPCR) with primers and probes targeting 3 genes (*ttr*, *staG* [STY0201], and *tviB*) was used to detect *S.* Typhi. Samples with all 3 targets present were considered positive for *S.* Typhi based on their demonstrated amplification in *Salmonella* species (*ttr*), Typhi and nontyphoidal *Salmonella* serovars (*staG*), and Typhi alone (*tviB*) [19]. Samples positive for *ttr* and either *staG* or *tviB* alone were retested in a singleplex qPCR for the negative target to improve the sensitivity of *S.* Typhi detection. We also conducted a duplex qPCR for HF183, a marker gene from a human-restricted *Bacteroides* [20], and an extraction/PCR positive control (Cy5-QXL670; Eurogentec) that was spiked into samples before extraction. Genome copy numbers were estimated based on standard curves generated using gBlocks DNA fragments for each target (Integrated DNA Technologies). The limit of detection (LOD) was estimated as the concentration that gave a positive result 95% of the time and genome copies calculated for samples above this threshold. Samples with a cycle threshold (Ct) value below the upper 95% confidence interval (CI) of the LOD or 40, whichever was lower, but above the point estimate for the LOD, were scored positive and given a genome copy number equal to the LOD.

### Typhoid Fever Hospital Surveillance

The number of blood culture-confirmed typhoid fever cases reported from tertiary and secondary hospitals serving the study population (including inpatients and outpatients, see [17]) were recorded during the period of ES. Patient age, date of culture, and location of residence were extracted for the analysis. These were compared with previous estimates of typhoid fever incidence in the study areas based on community surveillance (Vellore) and hospital cases (with and without adjustment for health care seeking and culture provision/sensitivity; Vellore and Blantyre) [21–23]. In Blantyre only, enhanced passive surveillance was conducted during the period of ES in 3 primary health care centers, including blood culture for children with febrile illness lasting ≥3 days [24].

### Statistical Analysis

The proportion of ES samples positive for *S.* Typhi (*ttr*, *staG,* and *tviB*) was calculated at each location for each sample type and 95% CIs calculated using mixed-effects logistic regression without covariates and a Gaussian random effect on the intercept to allow for repeat testing at each site. The intraclass correlation coefficient (ICC) was calculated from the maximum likelihood estimates of the fixed effect (intercept) and the variance of the random effect using Monte Carlo simulation [25]. Mixed-effects logistic and linear regression analysis was used to assess the association between detection of the different gene targets. Comparison of genome copy numbers for each target was based on rank correlation without correction for repeated measures because the relatively small number of samples positive for 2 or more targets did not allow the random effect by ES site to be estimated. The association of monthly ES detections in the study areas and clinical case numbers was assessed using binomial regression. Analysis was implemented in the R Statistical Programming language using the survey and ICCbin packages [26].

The crude incidence of culture-confirmed typhoid fever per 100 000 person years of observation in each location was calculated based on the number of cases in each age group recorded during the study period and the estimated population size within the study area. Exact 95% CIs were calculated based on the Poisson distribution. We did not estimate incidence in the community from these numbers using multipliers to adjust for health care seeking and culture availability [27]. This is because of the absence of contemporaneous health care utilization data and the likely large, uncertain adjustments that would be necessary during the coronavirus disease 2019 (COVID-19) pandemic when culture testing of febrile cases was severely limited.

### Ethical Approvals

The study protocol was reviewed by the ethics committee at Christian Medical College, Vellore (IRB 11170, OBSERVE) and College of Medicine Research Ethics Committee in Blantyre (COMREC P.07/0/3089).

## RESULTS

### ES Sites Selected and Sample Collection

A total of 40 and 43 ES sites, stratified into 3 location-specific catchment population sizes, were selected in Vellore and Blantyre, respectively (Table 1). Estimated catchment populations varied for both locations, ranging from 14 to 320 441 in Vellore and 1247 to 106 905 in Blantyre, with the catchments of 3 ES sites in Vellore extending beyond the targeted study area.

**Table 1.** Environmental Surveillance Site Characteristics

Characteristic	Catchment Classification		
	Small	Medium	Large
Vellore			
Number of sites	14	12	14
Median population (range)	233 (14–566)	1125 (750–4725)	28 036 (7119–320 441)
Median catchment area, km^2^ (range)	0.04 (0.01–0.14)	0.29 (0.1–144.8)	1.82 (0.09–177.9)
Median population density, number per km^2^ (range)	8121 (3939–12 932)	5076 (220–11 785)	7535 (401–337 525)
Blantyre			
Number of sites	15	14	14
Median population (range)	5961 (1247–8073)	17 451 (10 508–50 225)	80 935 (58 422–106 905)
Median catchment area, km^2^ (range)	0.8 (0.2–1.6)	2.6 (1.1–7.3)	18 (1.9–33.8)
Median population density, number per km^2^ (range)	6720 (4098–17 694)	6232 (3560–14 523)	4941 (3029–30 059)

In Vellore, 520 grab samples and 517 Moore swabs were collected between 3 May 2021 and 29 April 2022, corresponding to 12 monthly samples from all 40 ES sites with Moore swabs missing at just 3 sampling time points (across 2 sites). In Blantyre, 533 grab samples and 594 Moore swabs were collected between 4 May 2021 and 22 May 2022, with more variable sampling frequency reflecting intermittent challenges with access to sites (median 13 [range, 4 to 23] grab samples and 14 [range, 3 to 48] Moore swabs from 43 ES sites).

### Detection of *S.* Typhi in ES Samples

**Figure 1.**
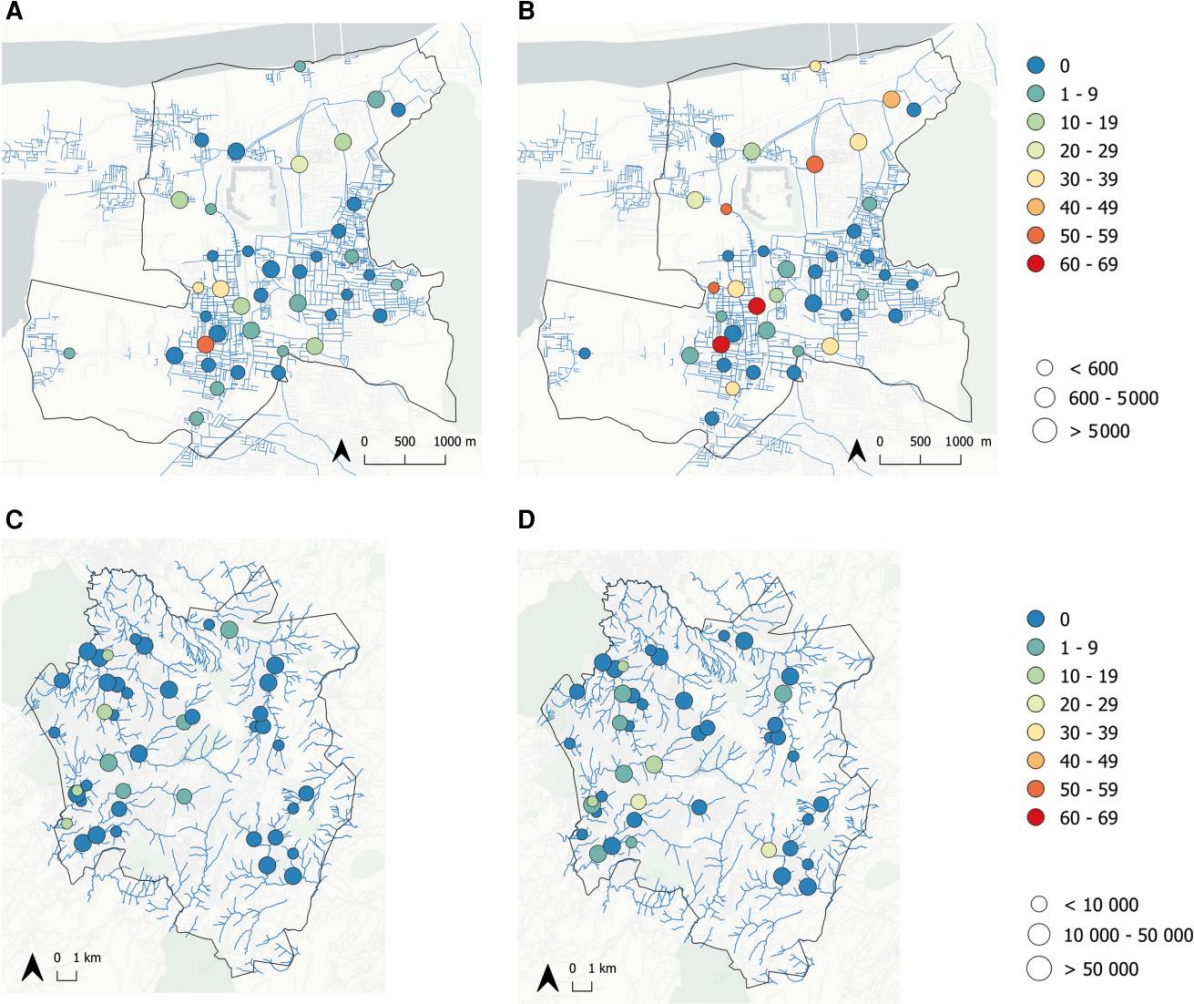
Location of environmental surveillance (ES) sites and the prevalence of *Salmonella* Typhi detection. ES sites are plotted as circles for Vellore (*A* and *B*) and Blantyre (*C* and *D*) with the shading indicating prevalence (percent of ES samples positive for *S*. Typhi) over the study period and results shown separately for grab samples (*A* and *C*) and Moore swabs (*B* and *D*). The radius of the circles is proportional to the estimated catchment population. Maps were created using QGIS version 3.22.4.

In Vellore, 39 (7.5%; 95% CI, 4.4%–12.4%) grab samples and 79 (15.3%; 95% CI, 9.8%–23.0%) Moore swabs tested positive for *S.* Typhi. Rates of detection were lower in Blantyre with 11 (2.1%; 95% CI, 1.1%–4.0%) grab samples and 23 (3.9%; 95% CI, 1.9%–7.9%) Moore swabs positive. The ICC was 0.11 and 0.29 for grab samples and Moore swabs in Vellore, and 0.02 and 0.06, respectively, in Blantyre. This indicates a moderate degree of clustering of detection of *S.* Typhi at specific ES sites in Vellore, as indicated on the map (Figure 1). The detection of *S.* Typhi in grab samples and Moore swabs was correlated with the estimated catchment population size for each ES site in Vellore (fixed effect of log number of people in mixed effects logistic regression was 0.622 and 0.944, respectively, *P* values .031 and .005). In Blantyre there was no significant association with estimated catchment population (*P* values = .760 and .445, respectively). The higher prevalence of detection of *S.* Typhi in Moore swabs compared with grab samples was significant in Vellore but not Blantyre (mixed effects regression *P* values <.001 and .158, respectively).

Detection of the *staG* gene target was correlated with the detection of the *tviB* target (ϕ coefficients of 0.71 and 0.61 for grab samples and Moore swabs, respectively, in Vellore [mixed-effect logistic regression test *P* values <.001]; 0.66 and 0.75, respectively, in Blantyre [*P* values <.001]; Table 2). Detection of the pan-*Salmonella* target *ttr* showed a modest association with detection of either *staG* or *tviB* (ϕ coefficients of 0.35 and 0.29 for these 2 targets in grab samples and 0.14 and 0.12, respectively, in Moore swabs in Vellore; 0.61 and 0.47 in grab samples and 0.70 and 0.57 in Moore swabs in Blantyre; mixed-effect logistic regression all *P* values <.05 except for the association of *ttr* with *staG* in grab samples in Vellore). Among those samples that were positive for all 3 targets, genome copy numbers inferred from the *tviB* and *staG* targets were significantly correlated (rank correlation coefficients 0.542 and 0.478, *P* values < .001 for grab samples and Moore swabs, respectively, in Vellore; 0.818 and 0.542, *P* values .004 and .009, in Blantyre; Supplementary Figure 1). Overall mean log genome copy number inferred for *Salmonella* species based on the *ttr* target were higher for Moore swabs compared with grab samples in Vellore (linear mixed effects regression, *P* value <.001; Table 2). There were no significant differences for the *staG* or *tviB* targets (*P* values .052 and .824, respectively). Fewer samples were positive in Blantyre and there were no significant differences in the copy numbers by sample type for any target.

**Table 2.** Combinations of *Salmonella* Typhi qPCR Targets Positive for Grab Samples and Moore Swabs in Vellore and Blantyre and the Mean Genome Copy Number

Sample Type		qPCR target detected			Mean log_10_ Genome Copy No. (Standard Error)		
	*ttr*	*staG*	*tviB*	Count	*ttr*	*staG*	*tviB*
Vellore							
Grab	Neg	Neg	Neg	259			
	Neg	Neg	Pos	3			0.3 (0)
	Pos	Neg	Neg	194	1.03 (0.03)		
	Pos	Neg	Pos	8	1.22 (0.2)		0.73 (0.07)
	Pos	Pos	Neg	17	1 (0.09)	0.7 (0.12)	
	Pos	Pos	Pos	39	1.14 (0.08)	0.75 (0.07)	0.87 (0.09)
Moore swab	Neg	Neg	Neg	37			
	Neg	Pos	Neg	1		0.34 (...)	
	Neg	Pos	Pos	1		0.82 (...)	0.84 (...)
	Pos	Neg	Neg	326	1.74 (0.04)		
	Pos	Neg	Pos	19	2.28 (0.17)		0.41 (0.06)
	Pos	Pos	Neg	54	2.4 (0.09)	0.89 (0.08)	
	Pos	Pos	Pos	79	2.37 (0.07)	0.91 (0.06)	0.91 (0.07)
Blantyre							
Grab	Neg	Neg	Neg	486			
	Neg	Neg	Pos	3			1.43 (0.21)
	Pos	Neg	Neg	25	1.31 (0.1)		
	Pos	Neg	Pos	2	1.11 (0.26)		1.99 (0.98)
	Pos	Pos	Neg	6	1.67 (0.2)	2.62 (0.16)	
	Pos	Pos	Pos	11	1.97 (0.21)	2.9 (0.17)	2.22 (0.13)
Moore swab	Neg	Neg	Neg	526			
	Neg	Neg	Pos	1			1.62 (...)
	Neg	Pos	Neg	1		2.6 (...)	
	Pos	Neg	Neg	30	1.19 (0.08)		
	Pos	Neg	Pos	1	1.21 (...)		1.11 (...)
	Pos	Pos	Neg	12	1.7 (0.15)	2.58 (0.09)	
	Pos	Pos	Pos	23	1.8 (0.11)	2.5 (0.19)	1.72 (0.16)

In Vellore, 514/520 (98.8%) grab samples and 515/517 (99.6%) Moore swabs were positive for the human-restricted *Bacteroides* HF183. In Blantyre, 345/533 (64.7%) grab samples and 279/594 (47.0%) Moore swabs were positive for HF183. Among those samples positive for HF183 in Blantyre, the prevalence of *S.* Typhi was 11/345 (3.2%; 95% CI, 1.7%–6.0%) and 19/279 (6.8%; 95% CI, 3.4%–13.2%) for grab samples and Moore swabs, respectively. Among these sample types negative for HF183, prevalence of *S.* Typhi was 0/188 (0.0%; 95% CI, 0.0%–1.9%) and 4/317 (1.3%; 95% CI, 0.5%–3.3%), respectively (*P* values for comparison with samples positive for HF183 in mixed effects logistic regression were .923 and .010, respectively). In Vellore, no samples negative for HF183 were positive for *S.* Typhi (0/8). The copy number of HF183 in ES samples from Blantyre was significantly higher among those that tested positive for *S.* Typhi (linear mixed-effects model *P* value = .001 for grab samples and <0.001 for Moore swabs).

**Figure 2.**
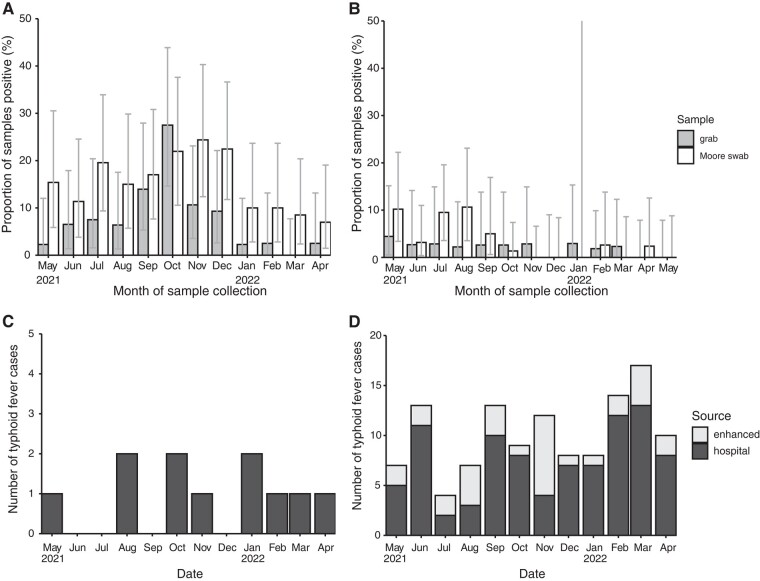
Monthly detection of *Salmonella* Typhi in environmental surveillance (ES) samples and the incidence of clinical cases reported from the study areas. The prevalence of *S.* Typhi across ES sites each month is shown for Vellore (*A*) and Blantyre (*B*) for each sample type. The error bars indicate 95% confidence intervals. The monthly number of blood culture-confirmed typhoid fever cases reported among inpatients and outpatients resident in the study areas from hospitals in (*C*) Vellore and (*D*) Blantyre. In Blantyre, additional cases detected through enhanced passive surveillance at 3 primary health care centers are also shown (gray).

Monthly prevalence of *S.* Typhi in ES samples varied during the study period in both locations (Figure 2). In Vellore, peak detection was observed in October and November 2021 for grab samples (27.5%) and Moore swabs (24.4%), respectively. In Blantyre, detection amongst Moore swabs was highest between May and August 2021 (3%–11%) whilst grab sample positivity remained low throughout the study (<5%).

### Incidence of Culture-Confirmed Typhoid Fever

Overall typhoid fever case numbers reported in hospitals serving the study areas during the period of ES were low (Table 3). Crude incidence of typhoid fever in Vellore and Blantyre was 7.8 (95% CI, 3.9–13.9) and 18.4 (95% CI, 14.8–22.6) per 100 000 people per year, respectively. The highest age-specific incidence was among children 0–4 years old (49.5 and 45.6 per 100 000 person years in Vellore and Blantyre, respectively). Inclusion of typhoid fever cases identified at primary health care clinics through enhanced passive surveillance in Blantyre increased overall incidence to 24.9 (95% CI, 20.7–29.7) per 100 000 person years, with peak incidence among children 5–9 years old (58.0 per 100 000 person years). Incidence of cases did not show a clear trend over time in Vellore or Blantyre (Figure 2). The monthly proportion of grab samples and Moore swabs positive for *S.* Typhi was not associated with the monthly incidence of typhoid fever in Vellore (binomial regression, *P* values = .649 and .513, respectively). In Blantyre, the monthly proportion of Moore swabs positive showed a negative association with typhoid fever incidence (*P* = .008 or .003 without or with cases from primary health care enhanced surveillance, respectively) but there was no association for grab samples (*P* = .705 or .841).

**Table 3.** Crude Incidence of Culture-Confirmed Typhoid Fever in Vellore and Blantyre, May 2021 to April 2022

Age Group, y	No. of Cases	Population	Crude Incidence Per 100 000 Person Years (95% CI)
Vellore			
0–4	4	8082	49.5 (13.5–126.7)
5–9	3	9772	30.7 (6.3–89.7)
10–14	0	10 430	0.0 (.0–35.4)
15–29	4	35 728	11.2 (3.1–28.7)
30+	0	77 788	0.0 (.0–4.7)
Total	11	141 800	7.8 (3.9–13.9)
Blantyre^a^			
0–4	30, 34	65 772	45.6 (30.8–65.1), 51.7 (35.8–72.2)
5–9	19, 40	68 946	27.6 (16.6–43.0), 58.0 (41.4–79.0)
10–14	14, 21	70 395	19.9 (10.9–33.4), 29.8 (18.5–45.6)
15–29	22	140 398	15.7 (9.8–23.7)
30+	5	144 397	3.5 (1.1–8.1)
Total	90, 122	489 908	18.4 (14.8–22.6), 24.9 (20.7–29.7)

^a^In Blantyre, enhanced passive surveillance of children with febrile illness was conducted at 3 primary health care centers serving the study area. Case numbers and incidence rates are shown without and with these additional pediatric cases (separated by a comma).

Abbreviation: CI, confidence interval.

## DISCUSSION

We present results from a comprehensive implementation of ES of *S.* Typhi in 2 cities with contrasting characteristics. *S.* Typhi was detected in multiple samples collected in Vellore, India and Blantyre, Malawi during 2021–2022 indicating continuous, local circulation. Prevalence in the environment was higher in Vellore compared with Blantyre, consistent with the higher incidence of clinical disease estimated in recent surveillance studies in these 2 locations. In Vellore, annual incidence was approximately 2000 cases per 100 000 children aged 0–14 years during 2017–2020 compared with approximately 700 during 2016–2018 in Blantyre, after adjusting for blood culture sensitivity, the probability of receiving a blood culture, and health care seeking [21, 22]. Prevalence in Vellore remained higher after adjusting estimates to include only samples with evidence for human fecal contamination (HF183).

During the study period, the number of typhoid cases reported in hospitals serving both study areas was substantially lower compared with previous years, particularly in Vellore where just 11 cases were reported compared with 98 during 2015 (meaning incidence was unexpectedly lower than Blantyre) [23, 28]. This may reflect genuine changes in transmission but also the impact of COVID-19 on health care seeking and provision of blood culture to febrile individuals. In the absence of contemporaneous health care utilization surveys and data on blood culture provision it is not possible to estimate the magnitude of this impact or whether COVID-19 has influenced *S.* Typhi transmission. The low case numbers reported during our study period meant we had limited power to detect an association between monthly ES detection of *S.* Typhi, and in Blantyre clinical incidence unexpectedly showed a modest negative association with *S.* Typhi detection in Moore swabs, but not grab samples. This may reflect differential effects of rainfall or other seasonal factors on ES sensitivity, or variability in health care seeking and testing during the single year of surveillance.

The detection of *S.* Typhi in Vellore showed moderate clustering by ES site with a higher prevalence of detection in the west and in northern sites draining from this community (Figure 1). ES sites with larger catchment populations in Vellore were also more likely to have *S.* Typhi detected. Overall prevalence of *S.* Typhi was lower in Blantyre and there was no evidence for clustering or association with catchment population.

Three gene targets were chosen to identify *S.* Typhi in ES samples. In brief, *ttr* is a pan-*S. enterica* gene, *tviB* is specific to *S.* Typhi and *S.* Paratyphi C, and *staG* is highly sensitive for *S.* Typhi but also found in nontyphoidal *Salmonella* serovars [19]. It is therefore reasonable to assume that co-occurrence of all 3 targets indicates the presence of *S.* Typhi. Although in theory an ES sample could be positive if it contained a mixture of *S.* Paratyphi C and nontyphoidal *Salmonella*, this is unlikely given the rarity of *S.* Paratyphi C [29].

Moore swabs deployed for 48 hours were more likely to result in the detection of *S.* Typhi compared with grab samples. The continued immersion of the swabs is likely to facilitate trapping of *S.* Typhi, which may be intermittently present in wastewater. Enrichment in UPE may also contribute to the enhanced sensitivity of the Moore swab protocol, which complicates interpretation of the inferred genome copy number. Grab samples have a theoretical advantage of allowing quantification of bacterial load by sample volume, but variable flow rates because of dilution by rainfall, uncertainty in catchment population, and more limited sensitivity suggest trap is preferable to grab samples for *S.* Typhi ES.

Our study was limited by the lack of information about community incidence of typhoid fever during the ES period and by inclusion of just 2 communities. This highlights the limitations of clinical surveillance, even in areas with support for hospital-based surveillance, because case numbers are strongly affected by health care seeking behavior, resources available for blood culture, and presumptive treatment with antibiotics. Estimates based on passive clinical surveillance need to be adjusted using imprecise and large multipliers, substantially inflating estimated incidence and the associated uncertainty (eg, 8.3 times in Malawi [27]). Triangulation of environmental and clinical surveillance with estimates of the incidence of infection based on seroprevalence surveys using *S.* Typhi-specific antigens may help decrease this uncertainty [30]. ES and serological surveillance capture asymptomatic as well as symptomatic infection and are independent of health care seeking. This contributes to the sensitivity of these surveillance mechanisms but may complicate comparison with clinical disease incidence and their interpretation by public health decision makers. Further work on the public health communication and utility of these novel surveillance mechanisms is needed.

Sensitivity of typhoid ES is likely to be affected by the characteristics of the wastewater network. Despite standardization of the site selection protocol, samples in Blantyre were by necessity collected from the river network or defunct sewage treatment works, whilst in Vellore they were mainly collected from open drains. This may result in differences in the probability of detecting fecal shedding of *S.* Typhi even after adjusting for human fecal contamination using HF183. Further work to identify determinants of ES site sensitivity based on site characteristics and water quality parameters is ongoing (cf [31]). The more frequent detection of *S.* Typhi at sites with larger catchment populations in Vellore suggests that existing polio ES sites, selected to represent populations >100 000 and implemented in many countries, may be leveraged to implement typhoid ES.

In conclusion, our data support the use of ES as an unbiased, low-cost tool to establish the local incidence of typhoid fever and to inform and monitor the introduction of TCV. Additional research sites with clinical surveillance programs (eg, Ghana, Nigeria, Fiji, Indonesia) are now implementing typhoid ES using standardized protocols to generate further data to support a potential scale-up that would help refine the world map of typhoid burden.

## Supplementary Data

Supplementary materials are available at *The Journal of Infectious Diseases* online (http://jid.oxfordjournals.org/). Supplementary materials consist of data provided by the author that are published to benefit the reader. The posted materials are not copyedited. The contents of all supplementary data are the sole responsibility of the authors. Questions or messages regarding errors should be addressed to the author.

## Supplementary Material

jiad427_Supplementary_Data

## Notes

***Financial support.*** This work was supported by the Bill and Melinda Gates Foundation (grant number INV-002381); the Medical Research Council (MRC) Centre for Global Infectious Disease Analysis (grant number MR/R015600/1), jointly funded by the UK MRC and the UK Foreign, Commonwealth and Development Office (FCDO), under the MRC/FCDO Concordat agreement; and the EDCTP2 program supported by the European Union. Funding to pay the Open Access publication charges for this article was provided through Jisc‘s ‘read and publish’ agreement with Oxford University Press.
