# Supplementary material for: Environmental Surveillance for Salmonella Typhi and its Association With Typhoid Fever Incidence in India and Malawi
Source: J Infect Dis. 2023 Sep 29;229(4):979–87. doi: 10.1093/infdis/jiad427 (PMC11011185; doi:10.1093/infdis/jiad427)
Supplement: jiad427_Supplementary_Data [file jiad427_supplementary_data.docx]

**Supplementary Figure 1**

A B


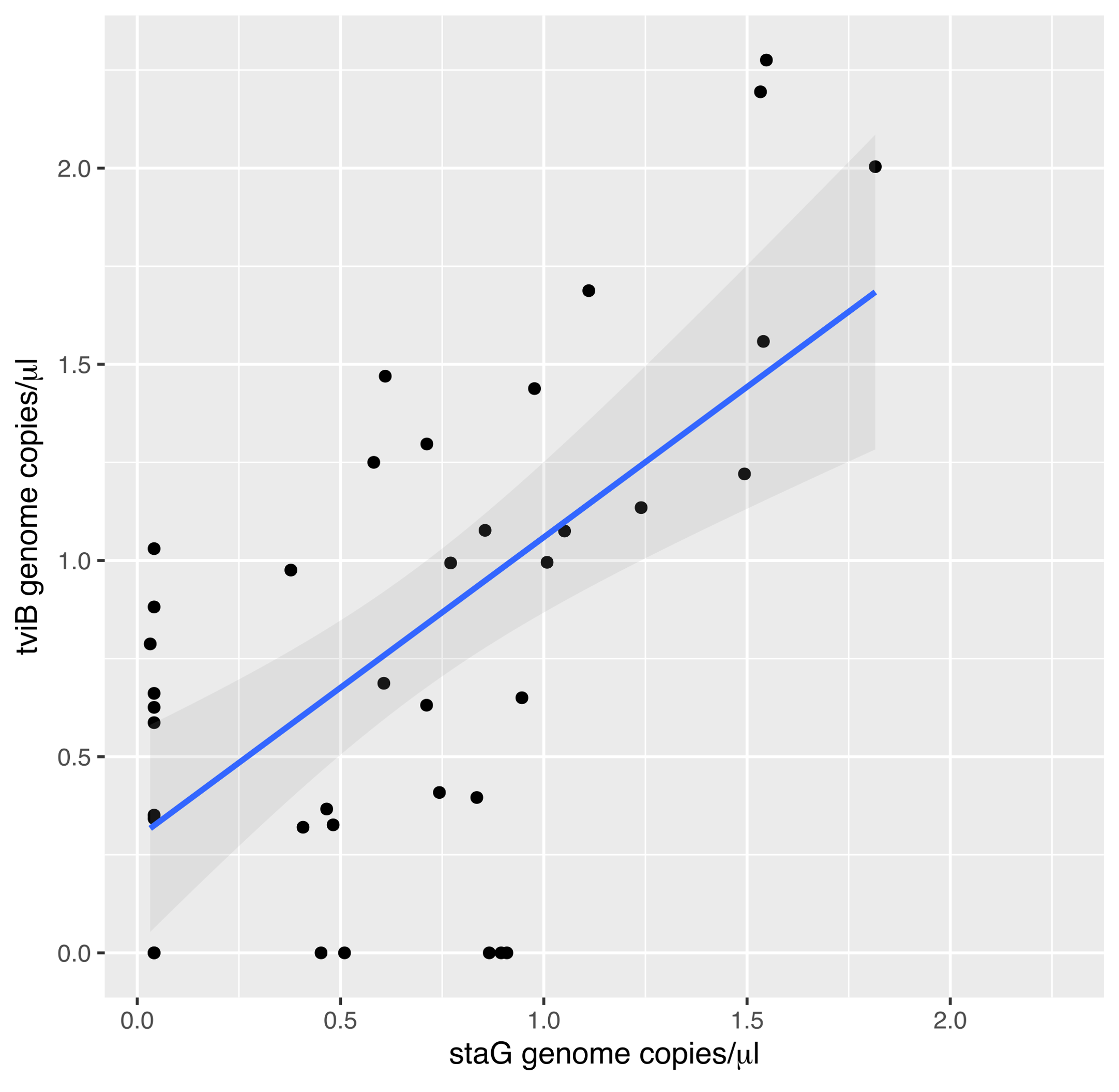

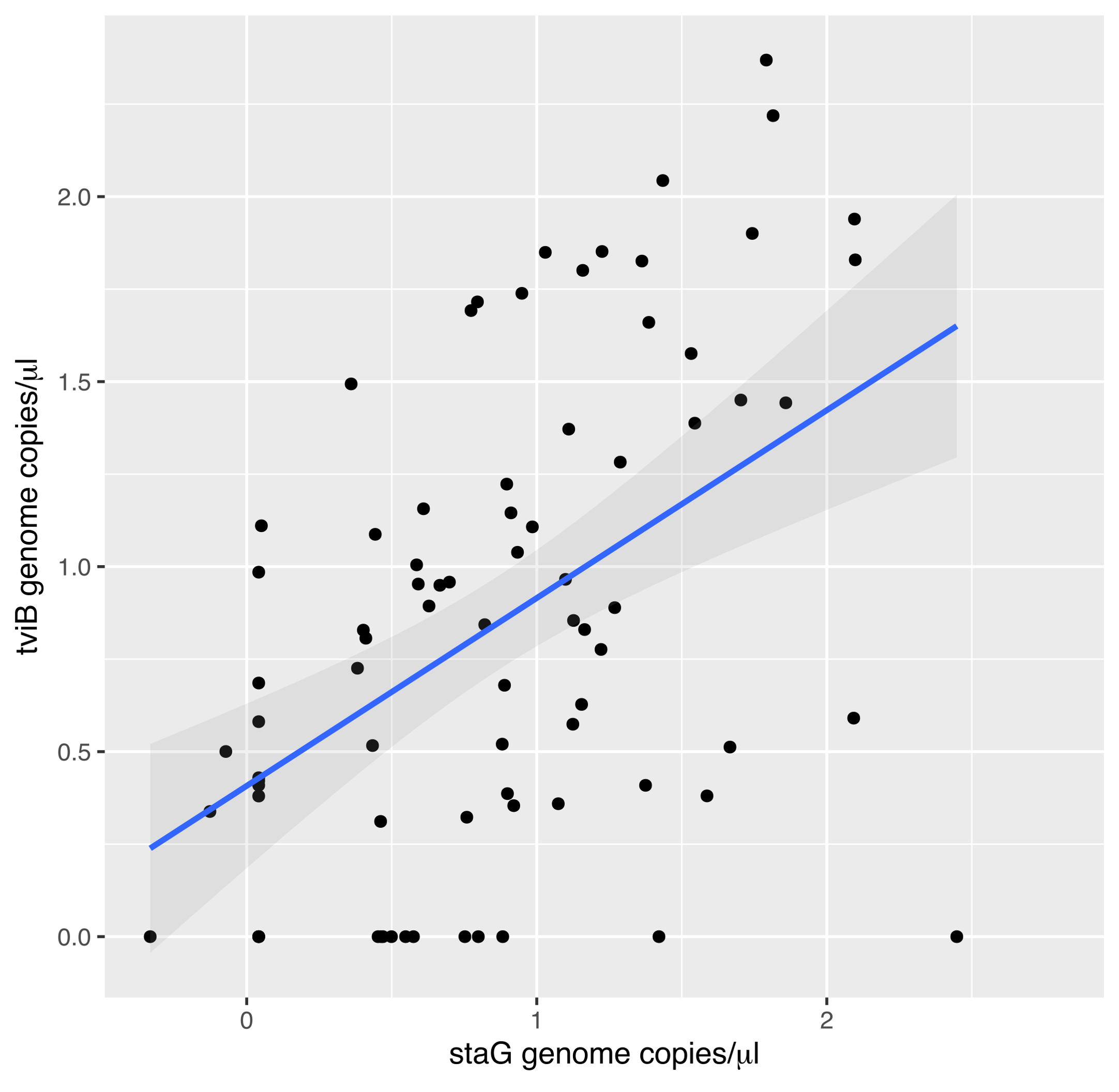


C D


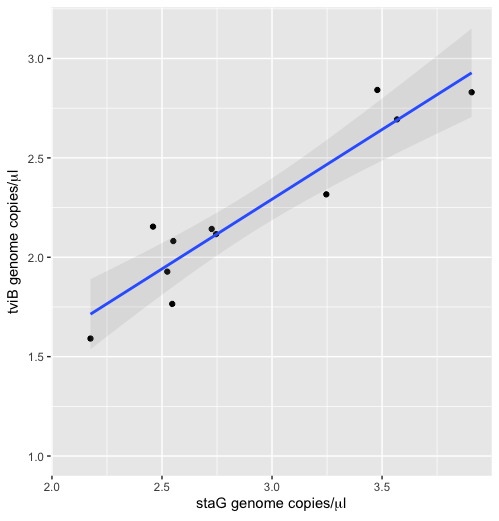

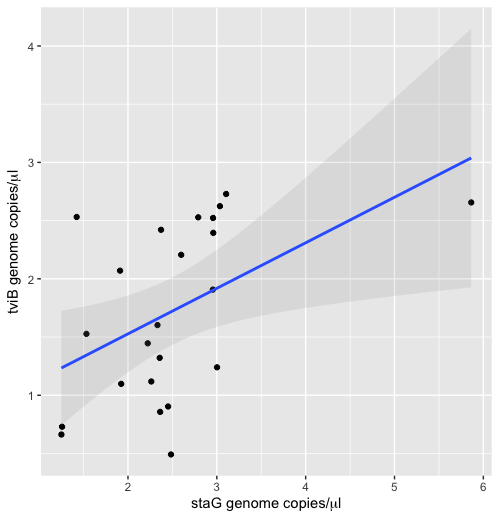


**Supplementary Figure 1** Estimated number of *S.* Typhi genome copies per μl DNA extraction based on the tviB compared with the staG gene target in Vellore (A,B) and Blantyre (C,D) for grab samples (A,C) and Moore’s swabs (B,D).
